# Variable Breeding Strategies in a Fluctuating Environment: A Feeding Experiment in Eastern Chipmunks

**DOI:** 10.1002/ece3.71076

**Published:** 2025-03-17

**Authors:** François Briau, Dany Garant, Denis Réale, Mathilde L. Tissier, Patrick Bergeron

**Affiliations:** ^1^ Département de Biologie Université de Sherbrooke Sherbrooke Quebec Canada; ^2^ Department of Biology and Biochemistry Bishop's University Sherbrooke Quebec Canada; ^3^ Département des Sciences Biologiques Université du Québec à Montréal Montréal Quebec Canada; ^4^ CNRS, IPHC UMR 7178 Université de Strasbourg Strasbourg France

**Keywords:** capital breeding, environmental cues, food availability, income breeding, plasticity, resource allocation, small mammals

## Abstract

Small mammals inhabiting pulsed‐resource environments, such as forests with intermittent seed masting of trees, often adjust their allocation to reproduction with the drastic fluctuations in food availability. They could, for instance, plastically allocate capital resources to reproduction when food availability is low or adopt an income breeding strategy when food availability is high. We investigated how eastern chipmunks ( *Tamias striatus* ), a food‐hoarding rodent strongly relying on the masting of beech trees ( *Fagus grandifolia* ), anticipate and respond to food pulses to reproduce. We supplemented a study site with sunflower seeds to buffer food availability for 2 years that were characterized by a non‐mast and a beech mast. We monitored chipmunks exploitation of the feeders, body mass, and reproductive activity. We tested if food‐supplemented females would reproduce in the absence of a beech mast. We compared those results with two control sites and 10 years of data on chipmunk reproduction. The probability of summer estrus significantly increased with the exploitation of feeders compared to the controls. Exploiting the feeders had a stronger effect than body mass on the probability of estrus. Still, beech masting had a broader effect on the probability of estrus across all sites than exploiting the feeders. Although most supplemented females showed oestrus during the non‐mast year, as opposed to non‐supplemented females, they did not fully reproduce despite their favorable body condition and the extensive hoard they accumulated. Those results suggest that summer reproduction in eastern chipmunks is not only financed by current food availability, revealing the complexity of plastic resource allocation in fluctuating environments.

## Graphical abstract

Observational studies provide limited information about the complex causality linking food anticipation, availability, and the reproduction of consumers. We conducted a feeding experiment on a free‐ranging population of eastern chipmunks, whose reproduction is tightly linked to the occurrence of beech masting events, and investigated how chipmunks anticipate food pulses to plastically allocate resources to reproduction.

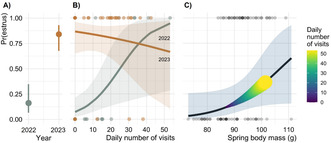

## 1 Introduction

Strategies of resource acquisition and allocation are central in shaping reproductive histories (Stearns 1992; van Noordwijk and de Jong 1986), and their expression often depends on cues associated with both environmental and body conditions (McNamara and Houston 1996; Rowe et al. 1994). For instance, most capital breeders in temperate regions, such as large mammals, reproduce seasonally by copulating in the fall to synchronize birth with optimal foraging conditions in the spring (Bronson 2009; Douhard et al. 2018). To achieve such timing, ovulation and spermatogenesis require a net positive energy balance, reached by the end of summer, and are triggered by photoperiod as a reliable environmental cue (Bronson 2009). In birds, temperature allows income breeders to time the hatching of their eggs with peak food abundance (Charmantier et al. 2008; Shutt et al. 2019; Visser et al. 2006). However, in fluctuating ecosystems with unpredictable patterns or multi‐annual cycles in resource availability, cues like photoperiod and temperature do not suffice to predict optimal conditions (Hart et al. 2020; Sarli et al. 2016; Schwimmer and Haim 2009). For example, the seed masting of dominant tree species can represent infrequent episodes of massive food availability, or food pulse, accompanied by an extended period of food scarcity (Cleavitt and Fahey 2017). Responses to more complex and reliable cues may allow organisms to cope with these unpredictable food pulses and provide further insights into understanding the complexity of energy acquisition and allocation to reproduction (Dore et al. 2018; Stephens et al. 2009, 2014; Williams et al. 2017).

Many species of small mammals are known to adjust their reproduction with drastic fluctuations in resource availability (Bergeron et al. 2011; Boutin et al. 2006; Lebl et al. 2010; Marcello et al. 2008; Schwimmer and Haim 2009). In temperate and northern forests, they appear to reproduce in anticipation of incoming food pulses of masting trees (Bergeron et al. 2011; Boutin et al. 2006; Lebl et al. 2010). For instance, red squirrels (*Tamiasciurus hudsonicus* and *Sciurus vulgaris* ) increase their reproductive effort in anticipation of white spruce ( *Picea glauca* ) masting events, potentially triggered by phytohormonal cues (Boutin et al. 2006; Dantzer et al. 2020; Petrullo et al. 2023). However, female squirrels are thought not to be limited by food availability, as they can rely on cached cones to invest in reproduction and would adopt such reproductive strategy to synchronize offspring recruitment with seed availability (Boutin et al. 2013; Dantzer et al. 2020). Similarly, in beech forests of Europe, the edible dormouse ( *Glis glis* ), a fat‐storing hibernator, only reproduces in anticipation of upcoming masting events and refrains from reproducing during non‐mast years. They synchronize lactation with the availability of energy‐rich beech seeds, which are thought to reduce the cost of reproduction for females and increase the survival of juveniles (Ruf and Bieber 2020). Lebl et al. (2010) suggested that food items early in the season, like flowers and unripe seeds, could act as a cue correlated with the upcoming masting events that could trigger a reproduction. The latter is consistent with the recent suggestion that organisms could exploit correlations in environmental variables to anticipate future conditions (see Bernhardt et al. 2020).

In the northern part of their range, Eastern chipmunks ( *Tamias striatus* ) also show anticipatory reproduction in the summer of masting years to synchronize the emergence of their juveniles with high beech seed availability in the fall (Bergeron et al. 2011). During a mast year, mating occurs around mid‐June and juveniles emerge from the maternal burrow in late August, as beechnuts become available (Tissier et al. 2020). How chipmunks finance the summer reproduction and the environmental cues they perceive to trigger ovulation remain unknown. Tissier et al. (2020) recorded a systematic diet shift toward red maple ( *Acer rubrum* ) seeds during the summer when chipmunks reproduce in anticipation of a beech mast, but the causal patterns explaining summer estrus and food availability have not yet been explored.

Here we conducted a supplemental feeding experiment on eastern chipmunks to improve our understanding of the cues that could allow small mammals to anticipate masting events and plastically fuel reproduction with capital resources. Our first objective was to test if food supplementing chipmunks could trigger a summer reproduction in the absence of a forthcoming beech mast. If true, the cue of summer reproduction would not be specific to beech trees, as previously hypothesized (Bergeron et al. 2011; Tissier et al. 2020). Because chipmunks hoard enormous amounts of food (Humphries et al. 2002) and since above‐ground activity is driven by food availability (Bergeron et al. 2011), our second objective was to evaluate the respective roles of food availability, food acquisition, and body mass on the onset of summer reproduction. We considered food availability as very high when feeders were available to chipmunks. We expected that our feeding experiment would result in a greater proportional increase in the food hoard size (exogenous capital) of an individual compared to its body mass (endogenous capital). Accordingly, we predicted the extent of feeder exploitation by an individual to predominantly influence its reproductive ‘decision’ over its increase in body mass. As food availability in late spring/summer could correlate with future environmental conditions, we also hypothesized that experimentally increasing food availability at that time could serve as an environmental cue, mimicking beech mast year spring conditions and misleading chipmunks to reproduce in anticipation of a beech mast.

## 2 Materials and Methods

### 2.1 Population and Data Collection

We monitored a population of eastern chipmunks in a deciduous forest of southern Québec, from 2013 to 2023 (45°05′ N, 72°26′ W). Our study area consisted of two 260 × 260 (sites 1 and 2) and one 180 × 180 trapping grid (site 3) located less than 10 km apart, a distance that limits habitat differentiation but prevents dispersal between sites. We used evenly distributed plastic buckets on all trapping grids under the canopy of mature trees to quantify seed production of beech and red maples (see Tissier et al. 2020 for more details). A year was considered a beech mast year when trees produced more than 50 seeds/m^2^. In early spring, chipmunks consume trout lilies ( *Erythronium Americanum* ) and spring beauties ( *Claytonia caroliniana* ), which are abundant herbaceous plants. Red maple seeds become available in June, while beech nuts are available in September. While chipmunks reproduced during the summer of beech mast years, they also reproduced in March of years following a beech mast, probably using the extensive hoards they accumulated during the previous fall. Those juveniles emerge in May, as the vegetative growing season begins (Bergeron et al. 2011).

Each trapping grid was marked at every 20 m. Trapping occurred systematically on every second coordinates (at 40 m distance) using Longworth traps checked at every 2–3 h. We usually trapped from 08:00 until dusk, from early May to late September. At first capture, chipmunks were marked with ear tags and subcutaneous PIT tags (Trovan Ltd.) for identification. PIT tags were also used to monitor access to the experimental feeders (see below). For each trapped individual, we recorded body mass using a 300 g Pesola spring scale, trap location, sex, and age (juvenile or adult). Individuals weighing less than 70 g at first capture and displaying no sign of sexual maturity (as darkened scrotum or apparent mammae) were considered juveniles; individuals weighing > 70 g were considered adults irrespective of their reproductive state. Females were considered to be attempting summer reproduction when captured with a swollen vulva (Smith and Smith 1975; Figure S1). Developed mammae and signs of lactation were considered effective reproduction (Bergeron et al. 2011; Tissier et al. 2020).

### 2.2 Food Supplementation

We began the feeding experiment in May 2022, a non‐mast year. Since we never recorded a summer reproduction occurring in the absence of a beech mast (Tissier et al. 2020), it was our first attempt to trigger a reproduction experimentally by adding food. We also provided food in 2023, a mast year. During both years, we provided chipmunks with ad libitum striped sunflower seeds on site 3 from May 19th to June 23rd, while the other two sites remained as un‐supplemented controls. The timing of the experiment was based on the usual summer pre‐reproductive period, which usually matches the timing of the red maple seeds availability during beech mast years (Tissier et al. 2020). We installed nine feeders (Figure S2) made of 208 L metal barrels fixed with straps to mature trees. Each feeder had two entrances made from 3.81 cm diameter ABS pipes. They were inserted on opposite sides of the barrels to avoid monopolization by individuals.

Chipmunk exploitation of the feeders was monitored using PIT tag RFIDLOG readers (Priority 1 Design, Melbourne, Australia) inserted inside the barrels. The readers were connected to circular antennas placed over the pipes where chipmunks reached the food (Figure S2). Antennas were in *pass‐by* mode (opposed to pass *through*) scanning from 05:00 to 21:00. We chose sunflower seeds because chipmunks are known to consume and store them (Bowers et al. 1993; Humphries, Kramer, et al. 2003). They also contain similar nutrients to beechnuts (Moore et al. 2022; Munro et al. 2005). Feeders were distributed 60 m from each other and evenly across the entire trapping grid (Figure S3), providing potential access to the resource to all individuals.

In 2022 and 2023, respectively, 69% and 84% of the individuals on the supplemented site had a PIT tag, for a total of 108,666 readings (55,282 in 2022; 55,384 in 2023). Since antennas can record many readings per second and chipmunks get in and out of a feeder during a single feeding spree, we pooled, as a single visit, the readings from an individual within a 2 min time interval (Figure S4–S6). This threshold was based on a preliminary assessment of the time needed for a chipmunk to store the acquired food at its burrow before returning to a feeder (unpublished data). We also used camera traps (Browning Trail Cameras, Alabama, U.S.A.) at the location of feeders to validate the readings and found a low antenna un‐detection rate of 1.4% (see Appendix S1). For each chipmunk with a PIT tag, we measured for both years: the number of feeders visited, the number of days with at least one visit, and the total number of visits. We also calculated the mean number of visits per day.

Animals were captured and handled in compliance with the Canadian Council on Animal Care, under the approval of the U. Sherbrooke Animal Ethics Committee (protocol number # 2019‐2182).

### 2.3 Statistical Analyses

We restricted our analyses to females because they are energetically more constrained than males with respect to reproduction and differ in how they allocate resources to reproduction (Hayward and Gillooly 2011; Trivers 1972; Williams et al. 2017). Compared to males, females usually display no signs of sexual activity during the summer of a non‐mast year (Table S1). We conducted analyses in two steps: first, we used both our long‐term database (2013–2021) and experimental data (2022–2023) to compare the natural trends in the reproductive activity of females with the patterns observed during the experiment. Second, we focused on the 2 years of the experiment alone to assess the effect of the feeders on the probability of summer estrus.

### 2.4 Feeding Experiment vs. Controls

We used all adult females captured from 2013 to 2023 to determine the number of females and the proportion of females in estrus each year for the three sites, given the natural fluctuations in food availability between years. Females not seen in estrus but with proofs of reproduction were implicitly considered to have been in estrus. Our goal was then to compare the probability of observing females in estrus on each site prior to 2022 and during the feeding experiment. We ran two generalized linear models (GLM) with a binomial error distribution on (1) the long‐term data from 2013 to 2021 (*n* = 451 obs.) and (2) on the experimental data in 2022 and 2023 (*n* = 107 obs.), to compare how the probability of estrus (no = 0; yes = 1) changed on the supplemented site compared to the control sites and the un‐supplemented years. In both models, we included sites and beech masting as fixed effects. GLMs were fitted in *R 4.3.1* using the *glm* function, and the probabilities returned by the models were extracted from the *sjPlot* package.

### 2.5 Effects of the Feeding Experiment

**FIGURE 1:**
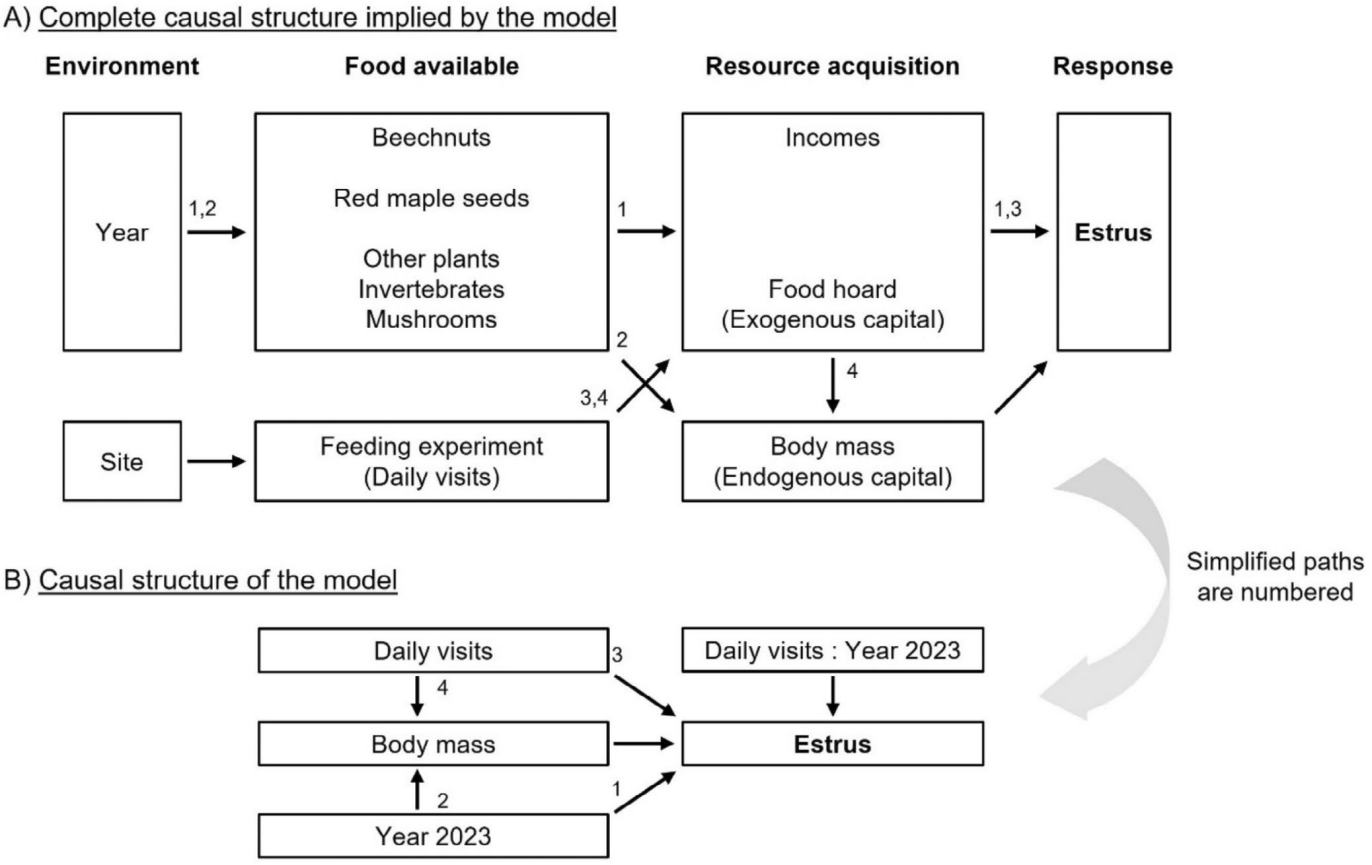
Simplification of the (A) complete hypothesized causal structure into (B) the causal structure of the piecewise structural equation model (pSEM). Simplified paths in (B) imply the causal structure in (A). Arrows represent unidirectional relationships between variables. We expected the environmental conditions to impact in cascades: Food availability, resource acquisition and the probability of summer estrus. In the model, daily visits were a proxy of food acquisition (hoard sizes). The model used the experimental data of 2022 and 2023 and included an interaction term between daily visits and year. The year 2022 was the reference.

We used the experimental data of 2022–2023 and a path analysis approach, favored for the study of causal relationships (Lefcheck 2016; Shipley 2016), to infer the potential determinants of summer estrus in female chipmunks. Based on a a priori knowledge of the study system and experimental design, we constructed a single structural equation model (SEM) to test our hypotheses (Tredennick et al. 2021). We aimed to translate our complete hypothesized causal structure through that model (Figure 1). We built the SEM using the R package *piecewiseSEM* (pSEM; Lefcheck 2016), a generalized form of the classical SEMs that uses a local instead of global estimation (i.e., a set of equations solved separately), permitting many family distributions (non‐normality and nonlinearity) and smaller datasets (Lefcheck 2016; Shipley 2000). The piecewise SEM integrated two response variables—*body mass* (linear regression) and *estrus* (logistic regression)—in a single causal structure (Figure 1B), which allowed us to interpret both the direct and indirect effects of the variables.

We expected spring *body mass* to have a direct effect on summer *estrus* and to be influenced by the feeding experiment and year. We expected *body mass* to mediate the indirect effects of these factors on summer *estrus*. Consequently, the piecewise SEM included (Figure 1B) the following explanatory variables: *year* (encompassing environmental conditions, beech masting and other unmeasured or unused food sources; see Figure 1A), the highest spring body mass recorded per female (hereafter referred as *body mass*, i.e., endogenous capital), and the mean number of visits per day at the feeders (*daily visits*) as a proxy for food acquisition (i.e., exogenous capital). We also added a *daily visits* × *year* interaction as we expected the feeding experiment to have a higher influence on summer *estrus* during the non‐mast year of 2022 than during the mast year of 2023. We did not include a site effect to avoid redundancy with the feeders being on one site. Individuals from the control sites were all given zero *daily visits* (not including those individuals as zeros in the analysis of Figure 4A provided the same biological conclusion, results not shown). By using the mean number of visits per day instead of the total number of visits during the experiment, we captured the intensity with which an individual was using the feeders in a single day. We did not include the identity of the individuals as a random effect since each chipmunk was sampled for 2 years maximum (Bolker et al. 2009). However, a test of directed separation confirmed that the identity did not explain variation in the probability of summer estrus when controlling for the *daily visits* and *body mass* of an individual (Table S2).

We used the standardized coefficients (*β*) of the piecewise SEM to compare the relative influence of each explanatory variable on the probability of summer estrus. The pSEM package also returned the proportion of variance explained by each explanatory variable (*R* ^2^‐values). For the body mass linear regression, we ran a Shapiro test to confirm normality (*p* = 0.79) and used the “DHARMa” and “Performance” packages to confirm homoscedasticity and non‐collinearity.

## 3 Results

### 3.1 Sites Comparison

**FIGURE 2:**
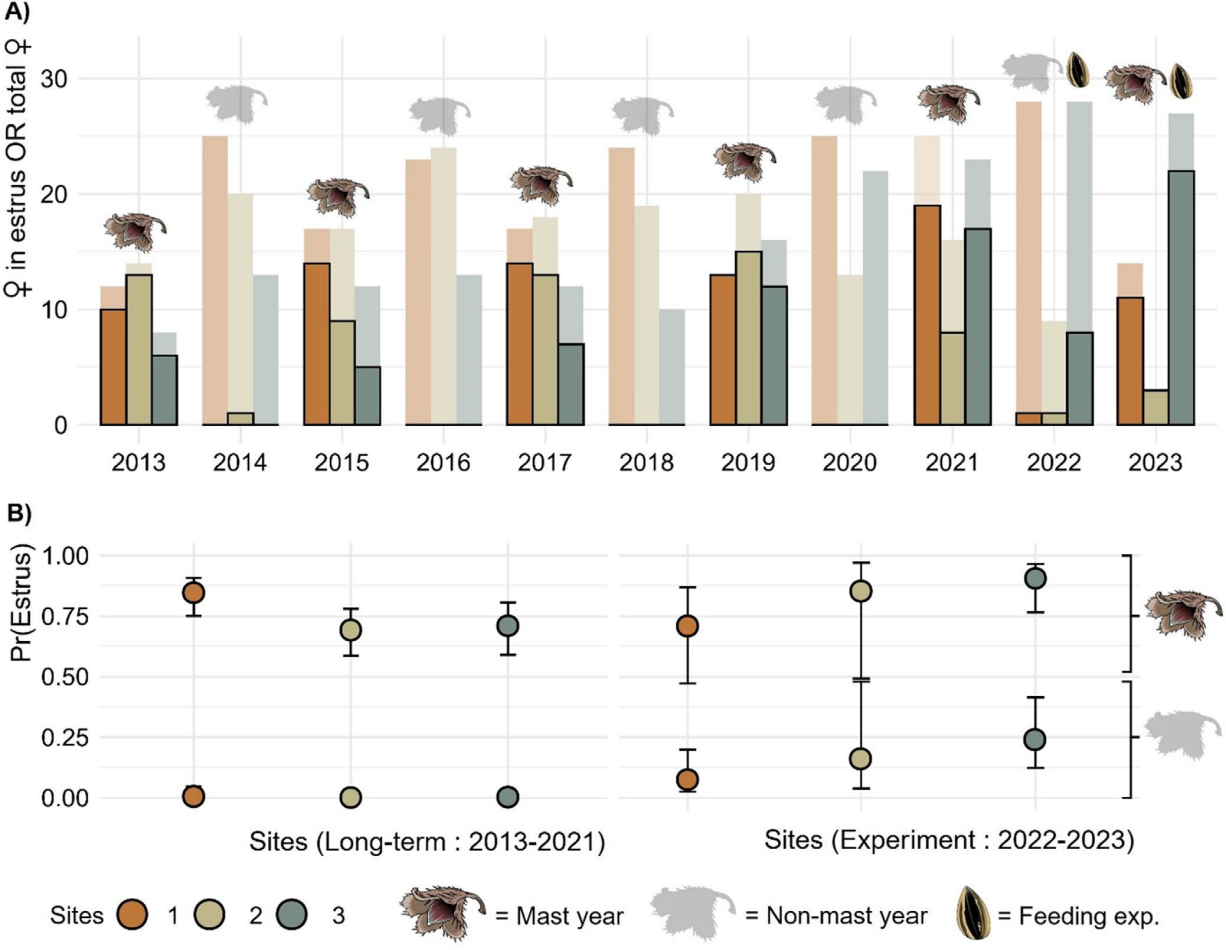
Female eastern chipmunks usually attempt summer reproduction during beech masting years only. (A) Number of females in estrus (dark colors) over the total number of adult females (pale colors) captured on each site from 2013 to 2023. (B) Long‐term and experimental probabilities of summer estrus among sites during mast and non‐mast years. Long‐term probabilities, *n* = 451. Experimental probabilities, *n* = 107 obs. Error bars represent 95% confidence intervals.

Before 2022, 99.5% of all summer estrus were recorded during mast years only (Figure 2A; Figure S7; Table S1). The model using long‐term data returned null probabilities of summer estrus during non‐mast years on all sites (Figure 2B; Table S3). Between 2013 and 2021, the probability of summer estrus during mast years was slightly higher on site 1 (0.85, CI [0.75, 0.91]) compared to sites 2 and 3 (respectively: 0.69, CI [0.59, 0.78]; 0.71, CI [0.59, 0.81]; Table S3). However, during the feeding experiment in 2022—a non‐mast year—28.6% of adult females displayed a summer estrus on the supplemented site 3 (Figure 2A; Table S1). The probability of summer estrus was significantly higher on the supplemented site in 2022 (0.24, CI [0.12, 0.42]) compared to the control sites (site 1: 0.07, CI [0.03, 0.2]; site 2: 0.16, CI [0.04, 0.48]; Figure 4B). Moreover, during the mast year 2023, the proportion of females in estrus on the supplemented site was 81.48%, the highest recorded on that site since 2013 (Figure 4A; Table S1). That year, the probability of summer estrus was still higher on the supplemented site (0.91, CI [0.77, 0.97]) compared to the control sites (site 1: 0.71, CI [0.47, 0.87]; site 2: 0.85, CI [0.49, 0.97]; Figure 4B; Table S3).

### 3.2 Feeding Experiment

**FIGURE 3:**
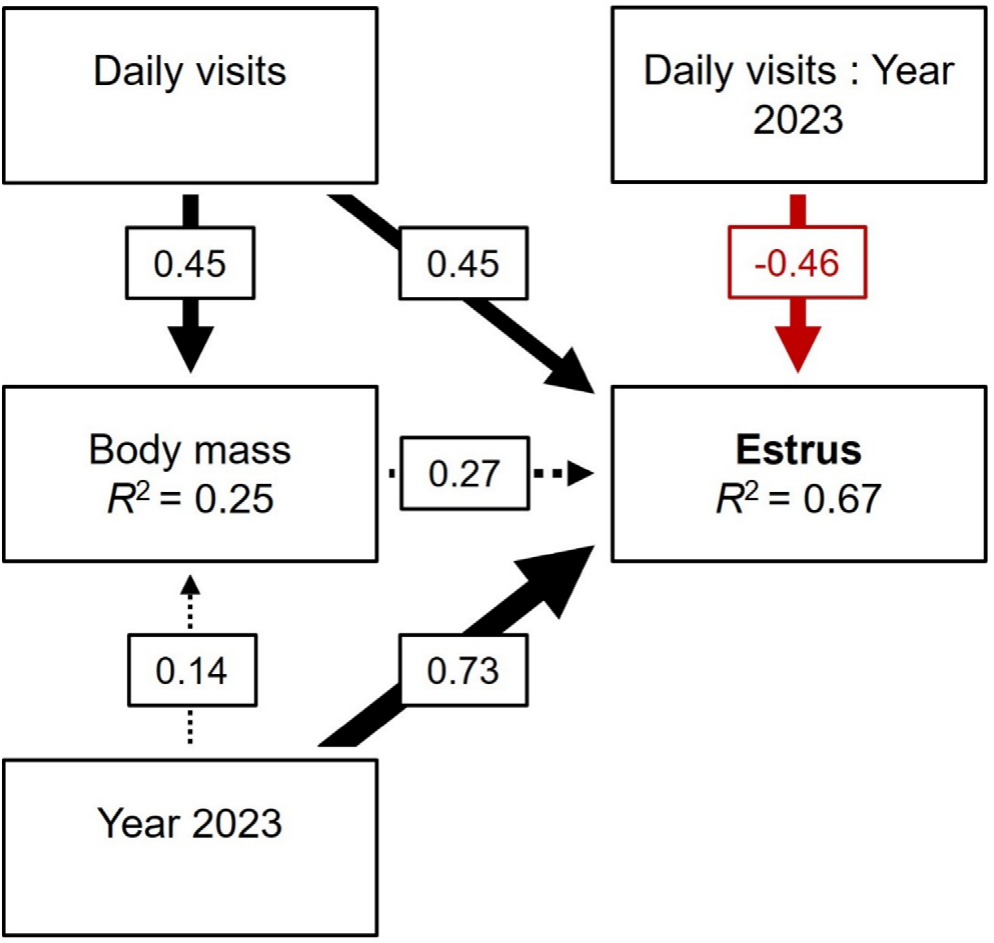
The feeding experiment (daily visits) increased body mass and the probability of summer estrus in female eastern chipmunks but its direct effect on estrus was dependent of the ecological context (non‐mast year 2022 and mast year 2023). The model included an interaction term between daily visits and year. Year 2022 was the reference. Arrows represent unidirectional relationships between variables. Black and red arrows denote positive and negative relationships respectively. The dotted arrows represent statistically nonsignificant paths (*p* > 0.05). Standardized coefficients (*β*) and arrows thickness represent the relative influence of each variable. Both response variables display a *R* ^2^ reflecting the proportion of variance explained by explanatory variables. *n* = 90 obs.

**FIGURE 4:**
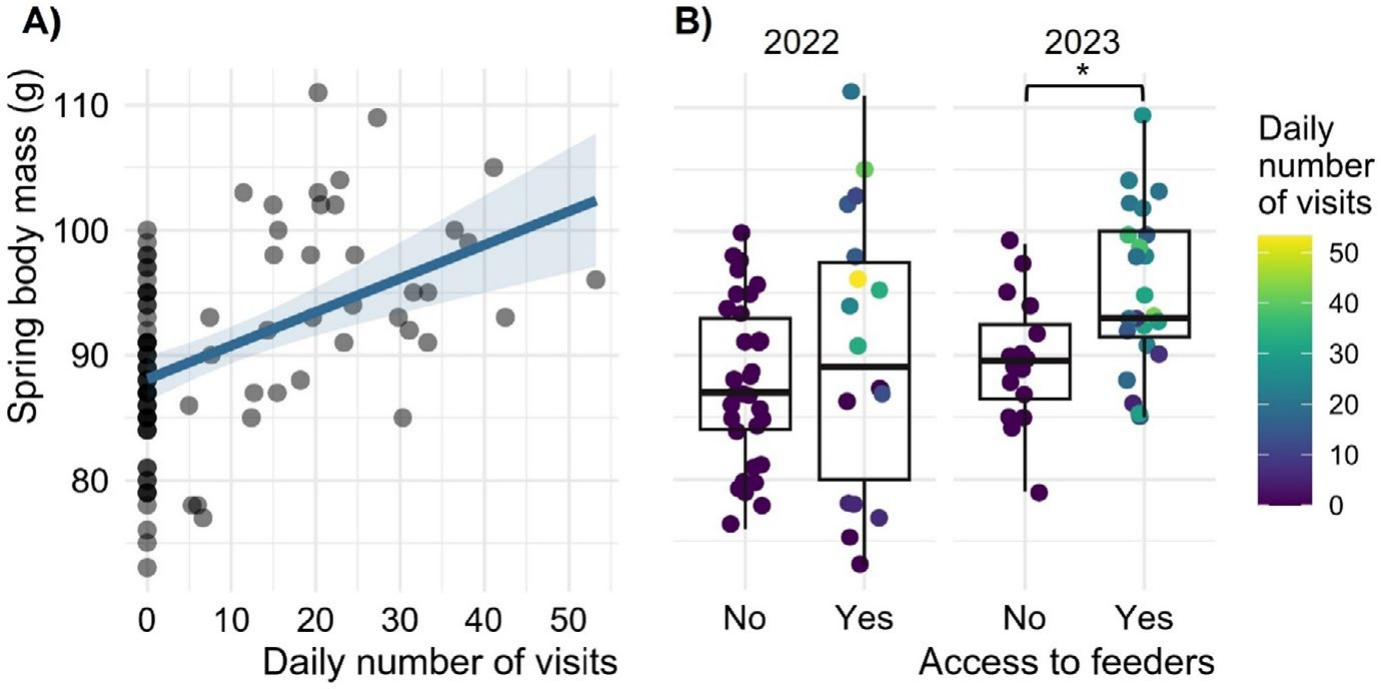
Effect of the feeding experiment on the spring body mass of female eastern chipmunks. (A) Highest spring body mass as a function of the daily number of visits to the feeders. (B) Boxplot of the highest spring body mass of individuals in 2022 and 2023 given access or not to the feeders. In (B), from left to right: *N* = 33/18/16/23 ♀. The asterisk denotes a significant difference between two groups (Wilcoxon test; *p* = 0.007). The shading in (A) indicates 95% confidence intervals.

The feeding experiment increased both *body mass* and the probability of summer estrus (Figure 3; Table S4). There was a significant positive effect of the *daily visits* on *body mass* (*p* < 0.001; Figure 3; Table S4) as females exploiting the feeders were heavier in late spring (Figure 4A). Accordingly, supplemented females tended to be heavier than controls (Figure 4B). However, the heaviest females were not necessarily those with the most visits at the feeders, as we observed great variability in *body mass* on site 3 for a given daily number of visits to the feeders (Figure 4B). The *year 2023* (compared to 2022) had no significant influence on *body mass* (2.2 ± 1.54 g; *p* = 0.16; Figure 3; Table S4), both in the presence and absence of feeders (Figure 4B). We found no significant difference in *body mass* between years for both the supplemented females (Wilcoxon; *p* = 0.16) and the controls (Wilcoxon; *p* = 0.39).

**FIGURE 5:**
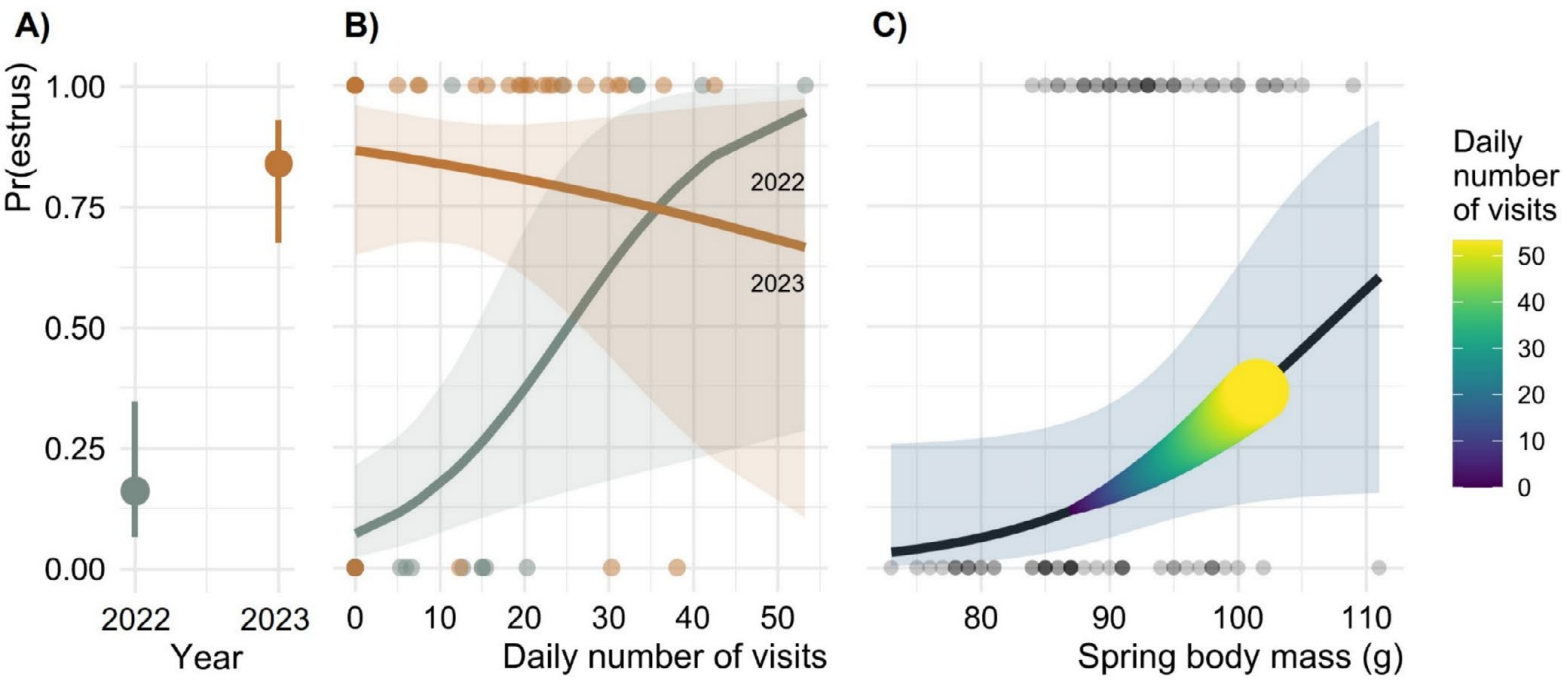
Probability of estrus in female eastern chipmunks as function of (A) year, (B) the daily number of visits to feeders for each year of experiment and (C) highest spring body mass, the latter being influenced by the daily number of visits to the feeders. In (B), the slopes for 2022 and 2023 are respectively 0.10 ± 0.04 and − 0.02 ± 0.05. The thickness of the curve and the color gradient in (C) reflect an increasing influence of the daily number of visits on the probability of estrus through spring body mass (indirect effect). Error bars in (A) and the shading in (B, C) indicates 95% confidence intervals. *n* = 90 obs.

During the non‐mast year 2022, the probability of estrus increased significantly with *daily visits* (Figures 3 and 5B; Table S4; *p* = 0.015). *Body mass* was also positively related to *estrus* (Figures 3 and 5C; Table S4; *p* = 0.061). When comparing the effect of both variables on *estrus* together, *daily visits* had a relatively larger influence than *body mass* (*β* = 0.45 vs. 0.27, respectively; Figure 3; Table S4). The overall effect of *daily visits* on *estrus* was even greater when considering the portion mediated by *body mass* (calculated by multiplying *β*_s_ as 0.45 * 0.27 = 0.12; the total influence of *daily visits* is then 0.45 + 0.12 = 0.57; the influence of *body mass* becomes 0.27–0.12 = 0.15; Figure 5C).

Since 43% of all adult females had access to the feeders in 2022 (Table S1), the probabilities of summer estrus returned by the model were 16% during the non‐mast year 2022 and 84% during the mast year 2023 at the population level (Figure 5A). The mast *Year 2023* had a higher influence than *daily visits* on the probability of *estrus* (*β* = 0.73; Figure 3; Table S4; *p* < 0.001). Given the *daily visits* × *year 2023* interaction, the direct influence of *daily visits* on *estrus* disappeared in 2023 (*β* = 0.45–0.46 = −0.01; Figures 3 and 5B; Table S4; *p* = 0.018).

## 4 Discussion

Providing unlimited food during a non‐mast and a mast year allowed us to study the interplay of energy acquisition and allocation for reproduction. Food acquisition had a stronger effect than body mass on the probability of estrus. Supplemented females increased in body mass, but those in estrus were not necessarily the heaviest. The effect of the feeders appeared to be context‐dependent, as the direct effect of food acquisition on the probability of estrus was high in 2022 (non‐mast year) but disappeared in 2023 (mast year). Only one supplemented female weaned juveniles in 2022 out of eight females who displayed estrus. In contrast, females from all sites produced juveniles in 2023.

### 4.1 Food Availability

The food acquired at the feeders had a predominant influence on reproduction compared to body mass, as the total effect of *daily visits* was 3.8 times higher than the direct effect of body mass (when attributing to *daily visits* the variation in *estrus* explained by its indirect effect through *body mass*). However, our model showed that exploiting feeders only directly influenced reproduction during the non‐mast year 2022, when food availability was naturally low (Gharnit et al. 2022; Tissier et al. 2020). During the mast year 2023, the proportion of females in estrus was high on all sites, and there was no direct effect of exploiting. Nonetheless, the indirect effect of exploiting feeders persisted through its effect on body mass, which itself had a moderate effect on the probability of estrus in both years. This suggests that lighter females were less likely to enter estrus and that supplemented females benefited from an alleviated trade‐off between maintenance and reproduction. Besides, supplemented females in 2023 were significantly heavier than controls, which may explain why we observed the highest proportion of females recorded in estrus on that site since 2013. Similar patterns were observed in Columbian ground squirrels ( *Urocitellus columbianus* ), an income breeder and fat‐storing hibernator, as females with the lowest body mass upon emergence in spring were unable to reproduce that year (Rubach et al. 2016). These unsuccessful females, however, gained more body mass during the active season than successfully breeding females, leading to the production of the largest litter size the following year (Rubach et al. 2016). In comparison, our results show that control females displayed a high proportion of estrus in 2023 without any significant change in body mass compared to 2022. Eastern chipmunks could benefit from maintaining a stable rather than plastic body mass across fluctuating environmental conditions (Nathoo et al. 2022). However, our results suggest that some somatic investment may also favor summer reproduction in chipmunks. Further investigations would be required on our supplemented site to assess if unused food in 2022 could have carried over to 2023 to increase body mass at spring emergence and facilitate summer reproduction.

Our findings are similar to what Lebl et al. (2010) recorded after experimentally feeding edible dormice, as food‐supplemented reproductive females were not heavier than non‐reproductive controls. Reproductive investment was independent of fat reserves. Like Columbian ground squirrels, dormice rely on fat stores for hibernation and adopt an income breeding strategy during the active season (Ruf and Bieber 2020). Yet, they time their reproductive activity with pulsed resources by using the abundance of rich food items in the spring as a signal of the upcoming seed production in the fall (Lebl et al. 2010; Ruf and Bieber 2020). Eastern chipmunks could adopt a similar strategy. However, since they rely on stored food during winter (Elliott 1978; Humphries et al. 2002), they can store more energy and manage it differently than fat‐storing hibernators (Humphries, Thomas, et al. 2003). In fact, free‐ranging chipmunks use their available energy reserves during winter to minimize torpor expression and its physiological costs (a trade‐off between their need to conserve energy and the risk of oxidative stress; Humphries, Kramer, et al. 2003; Munro et al. 2005). The use of torpor, mainly in early and late winter, could also vary among sexes and individuals under similar environmental conditions while being negatively associated with subsequent reproduction (Dammhahn et al. 2017). Moreover, higher plasticity in body mass has been associated with lower longevity and lower lifetime reproductive success in male eastern chipmunks (Nathoo et al. 2022), which suggests that seasonal fluctuations in body mass could be costly for females too.

### 4.2 Capital or Income Breeding Strategy?

Experimentally increasing food availability stimulated the expression of estrus at the population level. However, only one female did wean juveniles in 2022, while the others who displayed a summer estrus did not. Yet, they all had access to an unlimited food supply for 35 days. Considering that eastern chipmunks can load their cheekpouches with 4 g of sunflower seeds on average (Bowers et al. 1993), each reproductively active female may have consumed or accumulated kilograms of sunflower seeds during the supplementation. Supplemented females had higher body mass than average and were presumably not restricted by their food hoard after mating. It seems unlikely that reproduction was restricted by the number of reproductively active males (Table S1), nor by proteins, as one female still produced three healthy pups (unpublished data). Therefore, the apparent interruption of reproduction in 2022 may have followed the removal of feeders, as most females did not reproduce despite their stocks of sunflower seeds once the site returned to normal non‐mast year conditions. This suggests that, unlike the capital breeding strategy they display in spring, eastern chipmunks would not mainly rely on stored food to reproduce during the summer. Alternatively, summer reproduction may be driven by concurrent high food availability, and females could adopt an income breeding strategy, as reported for Siberian chipmunks ( *Tamias sibiricus* ) in a similar mast‐driven system (Coeur et al. 2018).

The absence of direct effect of exploiting feeders in 2023 compared to 2022 suggests that food availability was naturally high on all sites during that mast year (*before* the ripening of beechnuts), dampening the effect of additional food supply in sustaining an income breeding strategy. This hypothesis is supported by prior analyses of stable isotopes in our population, which revealed that chipmunks had a wider diet niche during beech masting years compared to non‐masting years (Gharnit et al. 2022). Moreover, seed production of many co‐occurring tree species could correlate with beech masting events, as interannual interspecific synchrony of seed production has been reported in many tree species (Gallego Zamorano et al. 2018; Koenig 2021; Schnurr et al. 2002; Shibata et al. 2002; Yang et al. 2020). Such temporal correlation in food availability, which appears to be a proximal cause of summer reproduction, could explain the predominant role of *Year* in our model (as hypothesized; Figure 1A) and why (as opposed to red maple seeds) beech seed production previously failed to be the best predictor of summer estrus in our system (Cinelli et al. 2022; Tissier et al. 2020).

### 4.3 The Cue of Summer Reproduction

Our results suggest that the initiation of summer reproduction in chipmunks would not rely on a beech‐specific cue but rather on high food availability, which, if temporally autocorrelated between spring and fall, could increase environmental predictability (Bernhardt et al. 2020) and potentially allow chipmunks to anticipate beech masting events. In red squirrels, females increasing their reproductive investment without an incoming food pulse have been reported to increase their lifetime reproductive success despite lower survival of juveniles (Petrullo et al. 2023). Interestingly, for such a species displaying an opportunistic reproductive strategy, missing a reproductive opportunity appears costlier than failing a reproductive attempt in poor conditions (Petrullo et al. 2023). In eastern chipmunks, Bergeron et al. (2011) reported that a cohort of summer‐born juveniles had suffered a low winter survival rate following a mast failure, but adults appeared unaffected. At the time, chipmunks were suspected of having misread the cue of masting. Considering our results, we can propose the alternative hypothesis that they did not misread the cue of a beech mast after all, but attempted reproduction given their perception of food availability and then failed to recruit their juveniles.

## 5 Conclusions

Our study supports the contention that fluctuating environments could drive adaptive plasticity in the allocation of resources (Williams et al. 2017), as chipmunks appear to use both capital and income breeding strategies, respectively, in spring and summer. Our feeding experiment was on a short time scale but is still ongoing, with the hope of further assessing chipmunks' reproduction over multiple years encompassing different sequences of mast and non‐mast years. For now, masting of beech trees seems to follow mostly a 2‐year cycle on our study sites. However, studies of seed production over longer terms show that a two‐year cycle is not the norm (Cleavitt and Fahey 2017). In addition, a wave of beech bark disease is currently decimating the beech trees on our sites, and we are thus in the front row of studying chipmunks reproductive dynamics through that unprecedented transition in the forest community. We are seeking the ecophysiological cues involved in triggering anticipatory reproduction and thus, we believe that long‐term studies of animals reproducing in pulsed systems are required to better understand how resource availability and predictability impact the adaptability of populations to reproduce in the context of global changes.

## Author Contributions

**François Briau:** conceptualization (lead), formal analysis (lead), methodology (lead), writing – original draft (lead), writing – review and editing (equal). **Dany Garant:** conceptualization (supporting), funding acquisition (equal), methodology (supporting), resources (equal), supervision (equal), validation (equal), writing – review and editing (equal). **Denis Réale:** conceptualization (supporting), funding acquisition (equal), methodology (supporting), resources (equal), supervision (supporting), validation (supporting), writing – review and editing (supporting). **Mathilde L. Tissier:** conceptualization (supporting), funding acquisition (equal), methodology (supporting), resources (equal), supervision (supporting), validation (supporting), writing – review and editing (supporting). **Patrick Bergeron:** conceptualization (supporting), funding acquisition (equal), methodology (supporting), resources (equal), supervision (equal), validation (equal), writing – review and editing (equal).

## Conflicts of Interest

The authors declare no conflicts of interest.

## Supporting information


Appendix S1.

## Data Availability

Analyses reported in this article can be reproduced using the data and code provided in Dryad at http://datadryad.org/stash/share/o9‐OlaS3PXN7PJDzpLWxkKlGU6e3lHBDKXuCpD1Abag.
