# Correction to “Variable Breeding Strategies in a Fluctuating Environment: A Feeding Experiment in Eastern Chipmunks”

**DOI:** 10.1002/ece3.72709

**Published:** 2025-12-15

Briau, F., Garant, D., Réale, D., Tissier, M.L. and Bergeron, P. (2025), Variable Breeding Strategies in a Fluctuating Environment: A Feeding Experiment in Eastern Chipmunks. *Ecology and Evolution*, 15: e71076. https://doi.org/10.1002/ece3.71076.

The following statement should be included in the Acknowledgments section:

“We acknowledge Allen Bush‐Beaupré for providing the codes used to visualize the statistical indirect effects (Figure 5c).”

We apologize for this error.

