# Supplementary material for: Variable Breeding Strategies in a Fluctuating Environment: A Feeding Experiment in Eastern Chipmunks
Source: Ecol Evol. 2025 Mar 17;15(3):e71076. doi: 10.1002/ece3.71076 (PMC11917122; doi:10.1002/ece3.71076)
Supplement: Supplementary file 1 — Appendix S1. [file ECE3-15-e71076-s001.docx]

Supplementary materials

Female summer reproduction


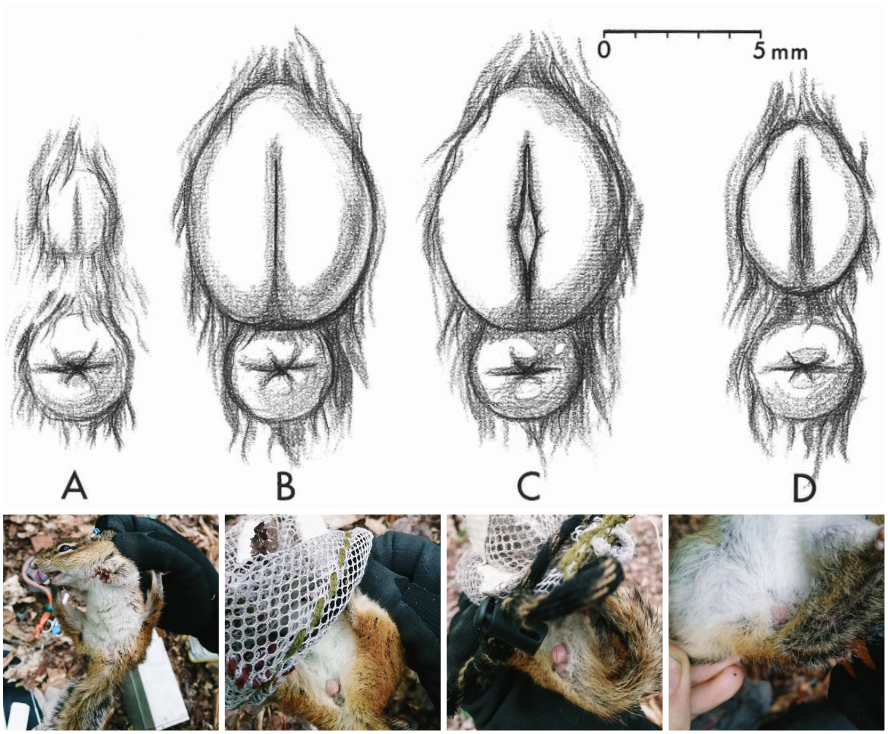


**Figure S1.** Drawing of external female genitalia of eastern chipmunks for (A) non-breeding, (B) early oestrus, (C) late oestrus, and (D) post-oestrus as depicted by Smith and Smith (1975), associated with pictures taken from the field of females at similar reproductive states.

**The feeders**

Feeders were designed to be resistant to weather and exploitation by non-targeted species, such as black bears (*Ursus americanus*), raccoons (*Procyon lotor*) and red squirrels (*Tamiasciurus hudsonicus*).


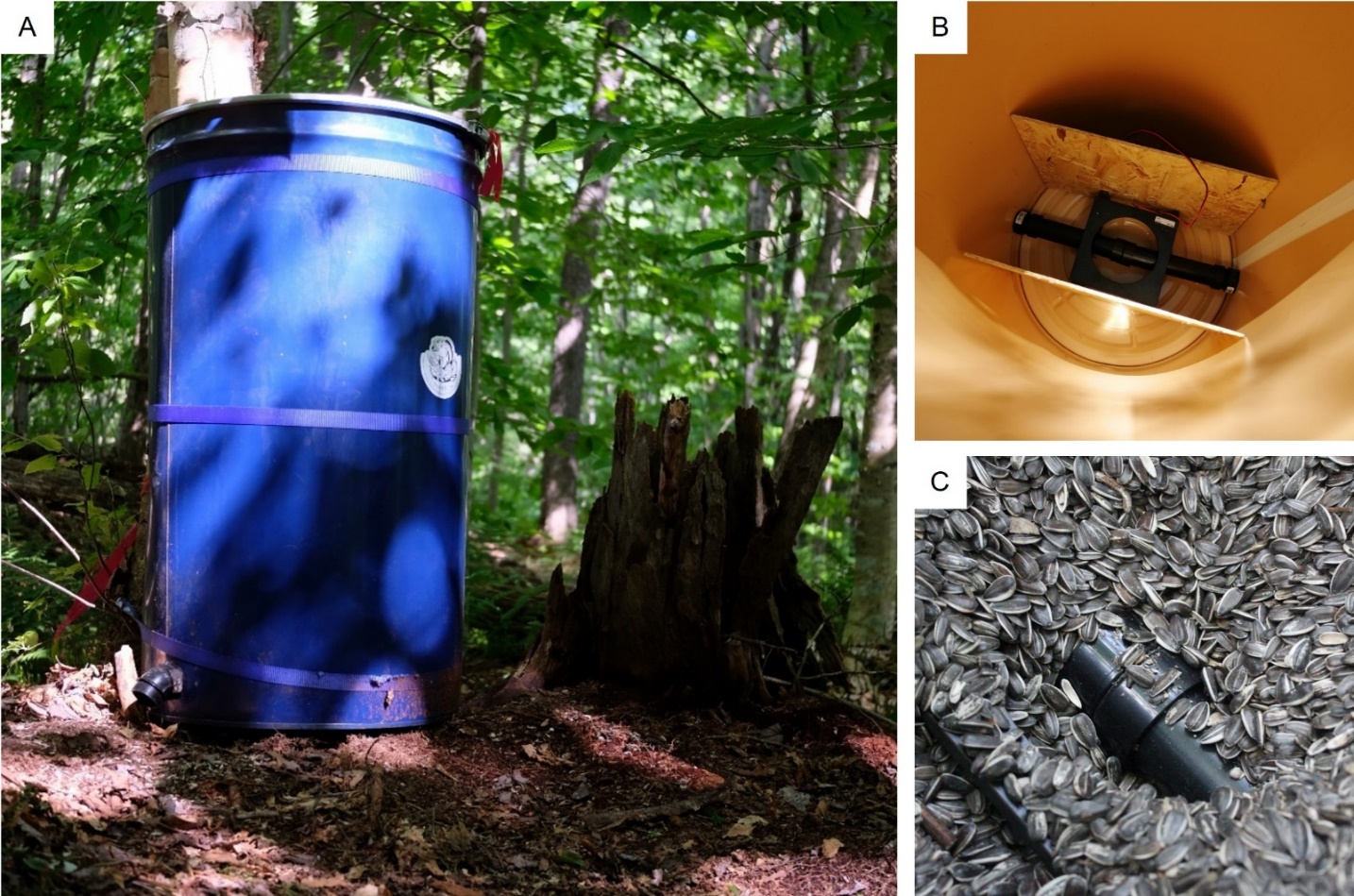


**Figure S2.** (A), barrel strapped to a mature tree. Only one door is visible to the left, the other is on the opposite side. PIT tag readers (not present on the pictures) are located inside the feeders. (B) Empty feeder with the two pipes capped under the antenna. We made a hole under the pipes to provide access to the food. (C) Sunflower seeds, as they are foraged, continuously refill the bottom area of the pipes.

**
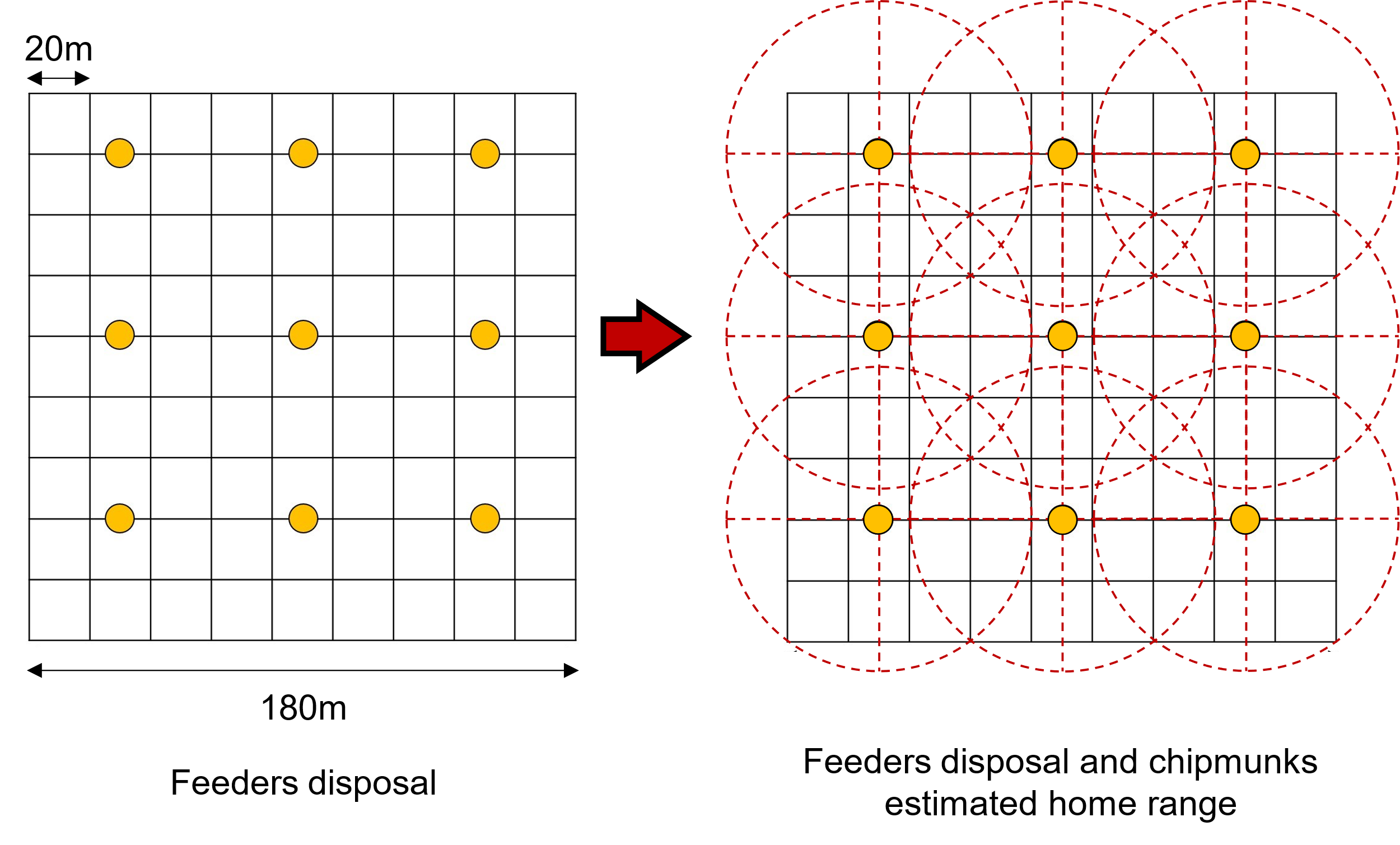
**

**Figure S3.** **The feeders were spread systematically across the trapping grid (180x180m) to cover site 3 entirely.** The red circles have a diameter of 100m, representing an approximation of the home range size of a chipmunk.

**Filtering the readings**

We pooled the readings with a time threshold interval of 2-min into a single visit considering that a major part of the readings would be actual noise (Fig. S4) and that travelling back and forth between a feeder and a burrow takes generally at least 2-min. Chipmunks enter a feeder many times at every visit to dispatch the sunflower seeds in their cheek pouches. Since we know the location of many burrows on our sites, selecting a 2-min period aimed at not underestimating the number of visits for individuals with burrows located close to the feeders. We tested many other time intervals (3 to 5-min). The counts of daily visits resulting from these tests were highly correlated (Fig. S5) and individuals, when compared, tended to keep their ranking (Fig. S6).


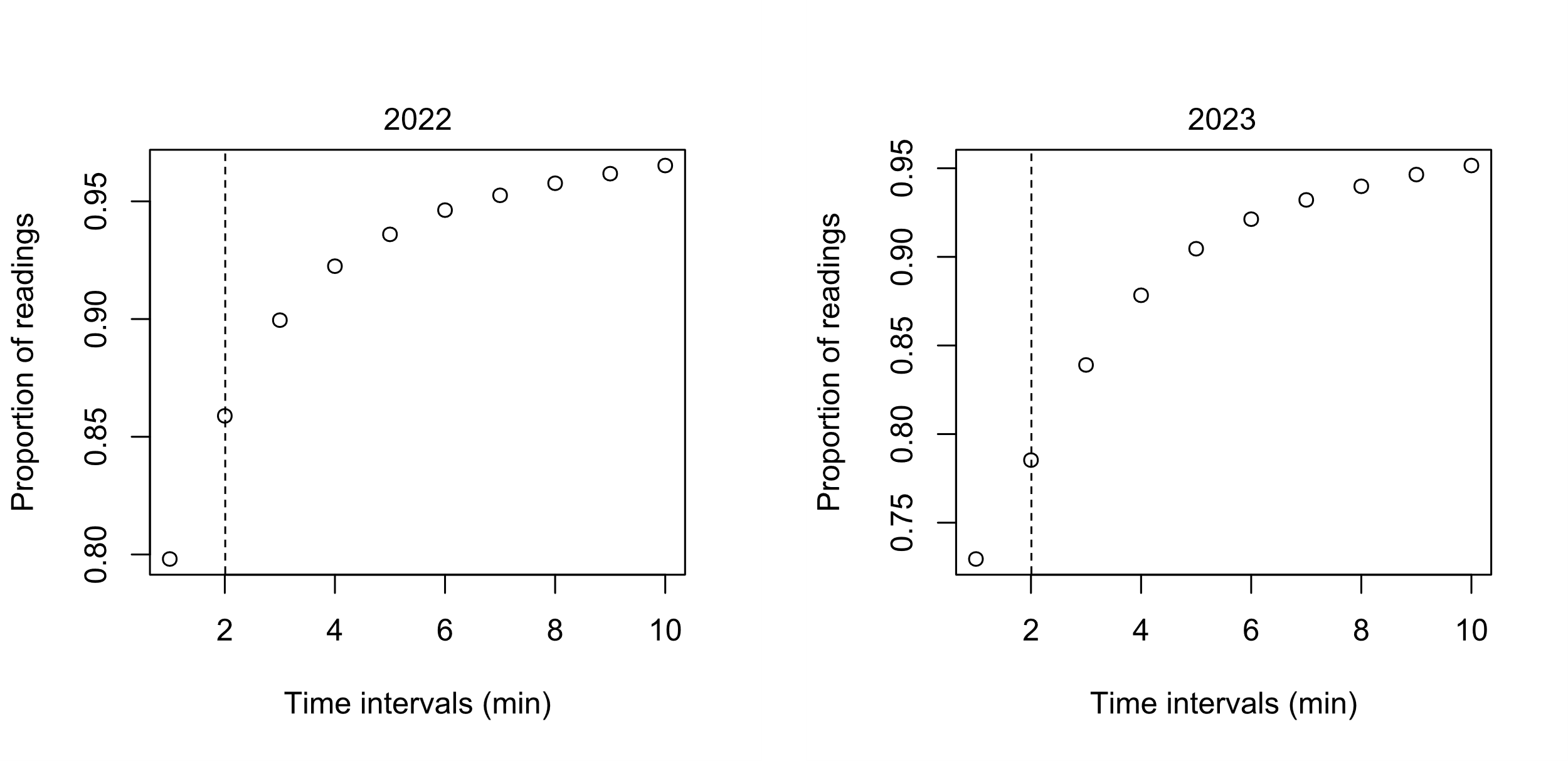


**Figure S4. Proportion of RFID readings filtered under various time intervals for 2022 and 2023.** For both years, more than 70% of the readings had an interval - between two visits for a same individual - shorter than 1-min. Those were considered as noise. The dotted line shows that by considering 2-min as our interval threshold, we eliminated around 80% of the readings. The remaining 20% were considered actual full visits at the feeders.


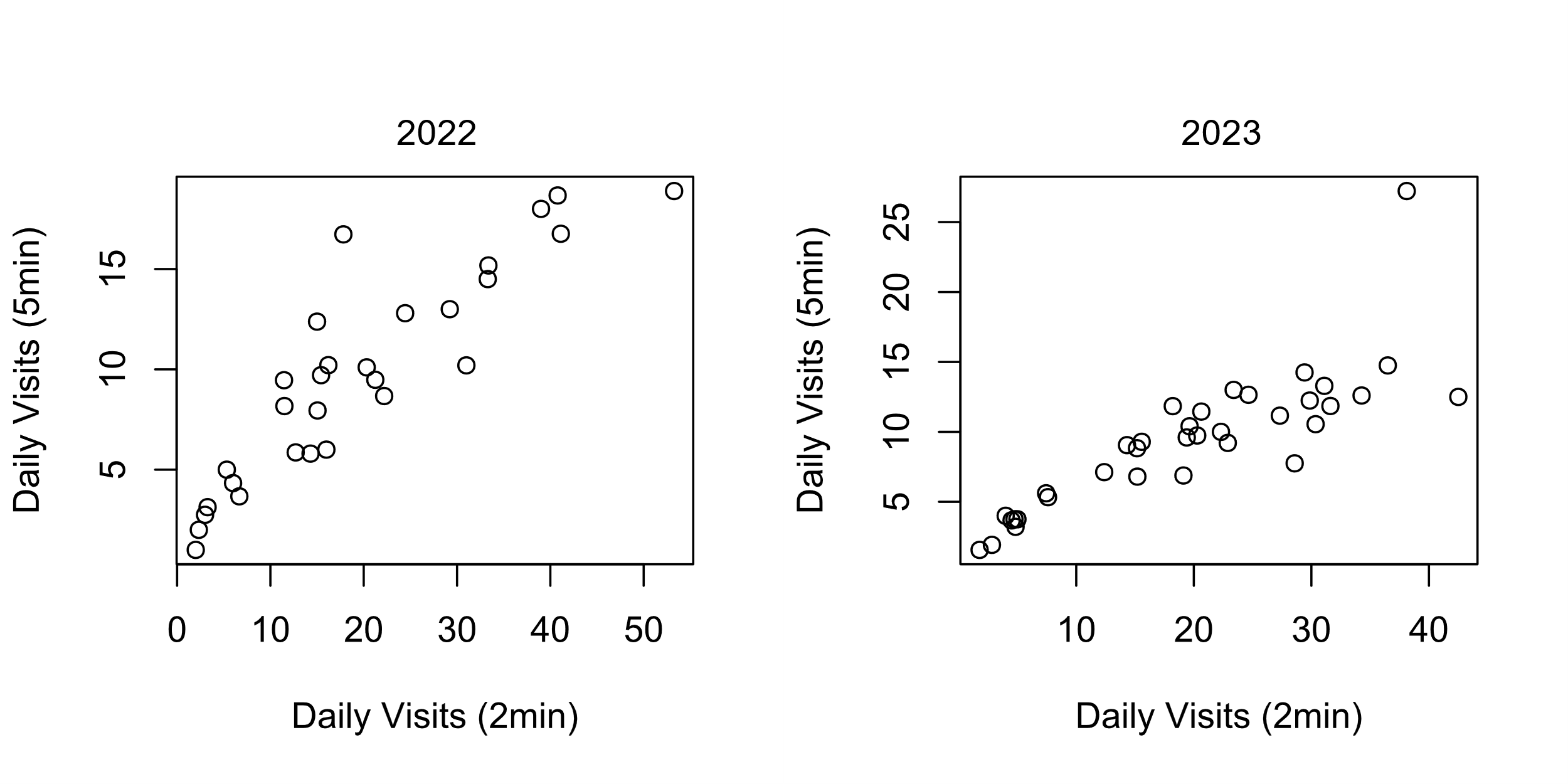


**Figure S5. The numbers of daily visits obtained using 2-min as an interval threshold for filtering RFID readings were highly correlated to the ones obtained using 5-min.** For 2022 and 2023 respectively: r = 0.90, p<0001; r=0.84, p<0.001.


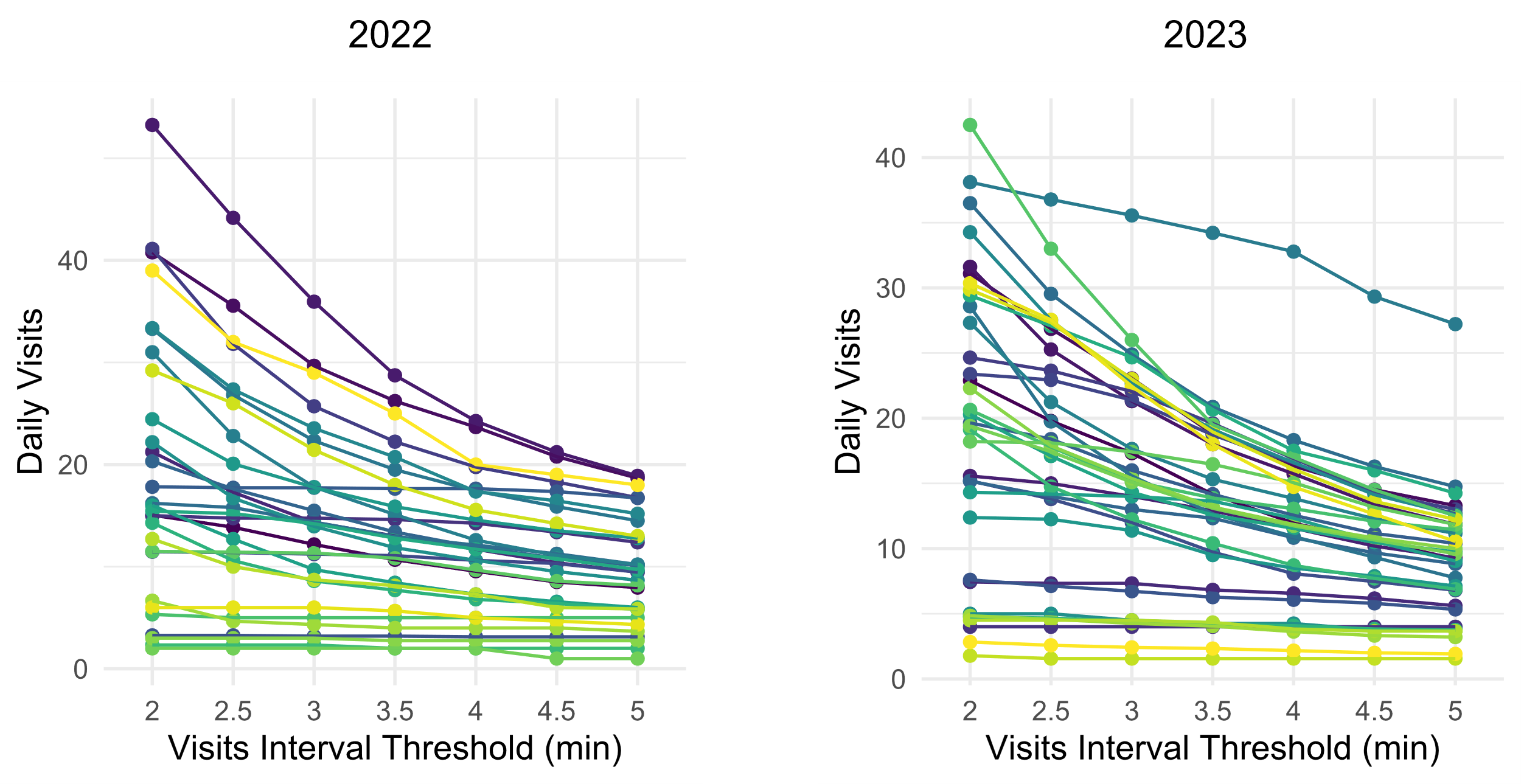


**Figure S6. Potential visits interval thresholds for filtering RFID readings were similar with lesser variation as the interval become longer.** Each color represents an individual and individuals tend to keep their overall ranking in the number of daily visits across the different intervals.

**Validation of the experimental design**

We tested our experimental design to validate the quality of our proxy of food acquisition. Calibrated camera traps (date and time) were set in front of every feeder to compare the readings of the PIT tag readers and the videos recorded. After analysing more than 5000 videos of 10 seconds, we used 727 videos displaying a chipmunk entering a feeder (in a pipe, where they can be detected by the antenna), we determined a proportion of concordance *reading-video* higher than the proportion of chipmunks with a PIT tag on the supplemented site (90%). The actual error rate we measured was of 1.4% but reached a maximum of 10% when considering uncertainty (i.e., considering individuals potentially not having a PIT tag, thus possibly representing false negative).

**Historical trend of reproductive activity during mast and non-mast years**

Table S1. Percentages (%) of adult males and females who displayed scrotal hypertrophy (SH) or estrus on each site from 2013 to 2023 given the masting cycle and the presence of feeders.

| Year | Masting | Site | No. ad. ♂ | No. SH | % SH | No. ad. ♀ | No. estrus | % Estrus | Feeders |
| --- | --- | --- | --- | --- | --- | --- | --- | --- | --- |
| 2013 | Mast year | 1 | 14 | 11 | 78.6 | 12 | 10 | 83.33 | No |
| 2013 | Mast year | 2 | 20 | 17 | 85 | 14 | 13 | 92.86 | No |
| 2013 | Mast year | 3 | 2 | 1 | 50 | 8 | 6 | 75 | No |
| 2014 | Non-mast year | 1 | 26 | 20 | 76.9 | 25 | 0 | 0 | No |
| 2014 | Non-mast year | 2 | 19 | 9 | 47.4 | 20 | 1 | 5 | No |
| 2014 | Non-mast year | 3 | 6 | 1 | 16.7 | 13 | 0 | 0 | No |
| 2015 | Mast year | 1 | 20 | 15 | 75 | 17 | 14 | 82.35 | No |
| 2015 | Mast year | 2 | 16 | 9 | 56.3 | 17 | 9 | 52.94 | No |
| 2015 | Mast year | 3 | 8 | 3 | 37.5 | 12 | 5 | 41.67 | No |
| 2016 | Non-mast year | 1 | 26 | 3 | 11.5 | 23 | 0 | 0 | No |
| 2016 | Non-mast year | 2 | 20 | 0 | 0 | 24 | 0 | 0 | No |
| 2016 | Non-mast year | 3 | 11 | 1 | 9.09 | 13 | 0 | 0 | No |
| 2017 | Mast year | 1 | 25 | 20 | 80 | 17 | 14 | 82.35 | No |
| 2017 | Mast year | 2 | 19 | 15 | 79 | 18 | 13 | 72.22 | No |
| 2017 | Mast year | 3 | 15 | 15 | 100 | 12 | 7 | 58.33 | No |
| 2018 | Non-mast year | 1 | 22 | 6 | 27.3 | 24 | 0 | 0 | No |
| 2018 | Non-mast year | 2 | 24 | 5 | 20.8 | 19 | 0 | 0 | No |
| 2018 | Non-mast year | 3 | 10 | 5 | 50 | 10 | 0 | 0 | No |
| 2019 | Mast year | 1 | 30 | 27 | 90 | 13 | 13 | 100 | No |
| 2019 | Mast year | 2 | 18 | 18 | 100 | 20 | 15 | 75 | No |
| 2019 | Mast year | 3 | 14 | 13 | 92.9 | 16 | 12 | 75 | No |
| 2020 | Non-mast year | 1 | 25 | 9 | 36 | 25 | 0 | 0 | No |
| 2020 | Non-mast year | 2 | 17 | 4 | 23.5 | 13 | 0 | 0 | No |
| 2020 | Non-mast year | 3 | 13 | 6 | 46.2 | 22 | 0 | 0 | No |
| 2021 | Mast year | 1 | 34 | 27 | 79.4 | 25 | 19 | 76 | No |
| 2021 | Mast year | 2 | 23 | 19 | 82.6 | 16 | 8 | 50 | No |
| 2021 | Mast year | 3 | 18 | 15 | 83.3 | 23 | 17 | 73.91 | No |
| 2022 | Non-mast year | 1 | 31 | 8 | 25.8 | 28 | 1 | 3.57 | No |
| 2022 | Non-mast year | 2 | 11 | 5 | 45.5 | 9 | 1 | 11.11 | No |
| **2022** | **Non-mast year** | **3** | 18 | 8 | 44.4 | **28** | **8** | **28.57** | **Yes** |
| 2023 | Mast year | 1 | 16 | 12 | 75 | 14 | 11 | 78.57 | No |
| 2023 | Mast year | 2 | 6 | 5 | 83.3 | 3 | 3 | 100 | No |
| **2023** | **Mast year** | **3** | 16 | 12 | 75 | **27** | **22** | **81.48** | **Yes** |

Legend: No. = number; % = percentage; ad = adults; SH = scrotal hypertrophy.

***
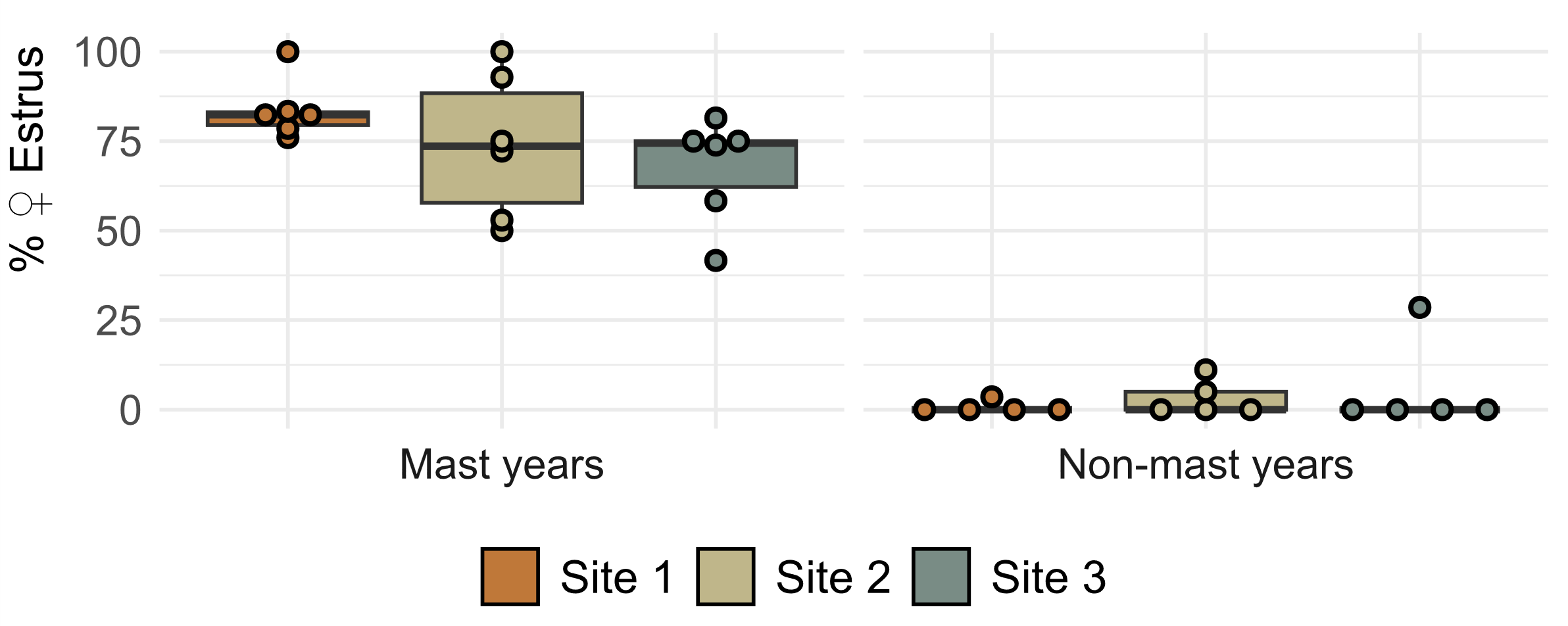
***

**Figure S7. Boxplot of the ratios of female eastern chipmunks recorded in estrus on each site during mast and non-mast years from 2013 to 2023.**

**Piecewise structural equation model and the test of directed separation**

The *piecewiseSEM* package provides, as a test of goodness-of-fit, the Bill Shipley’s test of directed separation (*d-sep test*). The latter procedure tests the assumption that all variables in the hypothesised causal structure are conditionally independent, i.e., that no relationship or causal path is ignored among unconnected variables (Lefcheck, 2016; Shipley, 2016). The package returned no goodness-of-fit for our piecewise SEM because all variables were already interconnected in our hypothesised causal structure. We were thus not missing any potential linkage between variables. However, we were unable to include the effect of the identity as random effect to control for the non-independence of our observations between 2022 and 2023 because each individual was sampled a maximum of two times (Bolker et al., 2009). By conducting a d-sep test, we verified that individuals were conditionally independent of summer estrus given the effect of *daily visits* and *body mass* (Table S2). The result of this test supports that *daily visits* and *body mass* translate very well the variation explained by the *identity* on the *estrus*, while, on the other hand, *identity* explains no left variation when controlling for *daily visits* and *body mass.* In other words, *identity* and *estrus* are only statistically linked to each other by mediators and no residual association exist between them after accounting for this mediating effect.

Table S2. Test of directed separation supporting that the effect of the individual is conditionally independent from the probability of summer estrus when controlling for the daily number of visits to the feeders and body mass. The p-value is over 0.9 for each ID. The statistically significant predictor (*p* < 0.05) is in bold.

| **Fixed effects** | **z.value** | **Pr(>\|z\|)** |  | **Fixed effects** | **z.value** | **Pr(>\|z\|)** |  | **Fixed effects** | **z.value** | **Pr(>\|z\|)** |
| --- | --- | --- | --- | --- | --- | --- | --- | --- | --- | --- |
| (Intercept) | -0.098 | 0.922 |  | ID#25 | -0.001 | 0.999 |  | ID#49 | 0.009 | 0.993 |
| ID#2 | 0.060 | 0.952 |  | ID#26 | 0.001 | 1.000 |  | ID#50 | 0.062 | 0.951 |
| ID#3 | -0.002 | 0.998 |  | ID#27 | 0.003 | 0.998 |  | ID#51 | 0.003 | 0.998 |
| ID#4 | -0.001 | 0.999 |  | ID#28 | 0.004 | 0.997 |  | ID#52 | 0.003 | 0.997 |
| ID#5 | 0.000 | 1.000 |  | ID#29 | 0.003 | 0.997 |  | ID#53 | 0.003 | 0.997 |
| ID#6 | 0.003 | 0.998 |  | ID#30 | 0.002 | 0.998 |  | ID#54 | 0.003 | 0.998 |
| ID#7 | 0.003 | 0.997 |  | ID#31 | -0.002 | 0.998 |  | ID#55 | 0.003 | 0.998 |
| ID#8 | 0.006 | 0.995 |  | ID#32 | 0.003 | 0.998 |  | ID#56 | 0.003 | 0.997 |
| ID#9 | 0.005 | 0.996 |  | ID#33 | 0.005 | 0.996 |  | ID#57 | 0.000 | 1.000 |
| ID#10 | 0.061 | 0.951 |  | ID#34 | -0.005 | 0.996 |  | ID#58 | 0.005 | 0.996 |
| ID#11 | 0.003 | 0.998 |  | ID#35 | 0.061 | 0.951 |  | ID#59 | -0.003 | 0.998 |
| ID#12 | 0.003 | 0.998 |  | ID#36 | 0.000 | 1.000 |  | ID#60 | 0.002 | 0.999 |
| ID#13 | -0.012 | 0.991 |  | ID#37 | 0.003 | 0.998 |  | ID#61 | 0.001 | 0.999 |
| ID#14 | 0.059 | 0.953 |  | ID#38 | 0.000 | 1.000 |  | ID#62 | 0.005 | 0.996 |
| ID#15 | 0.003 | 0.998 |  | ID#39 | 0.003 | 0.998 |  | ID#63 | 0.000 | 1.000 |
| ID#16 | 0.057 | 0.955 |  | ID#40 | 0.003 | 0.998 |  | ID#64 | -0.001 | 0.999 |
| ID#17 | 0.059 | 0.953 |  | ID#41 | 0.003 | 0.998 |  | ID#65 | -0.003 | 0.998 |
| ID#18 | 0.004 | 0.997 |  | ID#42 | 0.003 | 0.998 |  | ID#66 | 0.003 | 0.998 |
| ID#19 | 0.059 | 0.953 |  | ID#43 | 0.063 | 0.949 |  | ID#67 | 1.211 | 0.226 |
| ID#20 | 0.004 | 0.997 |  | ID#44 | 0.060 | 0.952 |  | ID#68 | 1.961 | 0.050 |
| ID#21 | 0.003 | 0.998 |  | ID#45 | 0.048 | 0.961 |  | ID#69 | 0.005 | 0.996 |
| ID#22 | 0.003 | 0.998 |  | ID#46 | 0.001 | 1.000 |  | Daily visits | 1.211 | 0.226 |
| ID#23 | 0.060 | 0.952 |  | ID#47 | 0.003 | 0.998 |  | **Body Mass** | 1.961 | **0.050** |
| ID#24 | 0.003 | 0.998 |  | ID#48 | 0.002 | 0.999 |  |  |  |  |

Results from the models

Table S3. Long-term (2013 to 2021) and experimental (2022 and 2023) coefficient estimates for female eastern chipmunks’ summer estrus, function of the site and the beech masting cycle (mast vs non-mast years). Sample sizes are 451 obs. (252♀) and 107 obs. (77♀) for the long-term and experimental models respectively. Results are from two distinct logistic regressions using the R glm function.

|  |  | **Estimate** | **SE** | **z value** | **Pr(>\|z\|)** |
| --- | --- | --- | --- | --- | --- |
| **Long-term model** | Site 1 | 1.702 | 0.303 | 5.625 | 0.000 |
|  | Site 2 | -0.893 | 0.381 | -2.345 | 0.019 |
|  | Site 3 | -0.808 | 0.404 | -2.003 | 0.045 |
|  | Non-mast | -6.689 | 1.024 | -6.535 | <0.001 |
| **Experimental model** | Site 1 | 0.887 | 0.511 | 1.736 | 0.083 |
|  | Site 2 | 0.865 | 0.938 | 0.922 | 0.357 |
|  | Site 3 | 1.366 | 0.612 | 2.232 | 0.026 |
|  | Non-mast | -3.407 | 0.579 | -5.885 | <0.001 |

Table S4. Results from the piecewise structural equation model (pSEM). The two response variables are separated by a dotted line. The year of reference is 2022. Statistically significant predictors (p < 0.05) are in bold. Estrus: R^2^=0.68. Body mass: R^2^=0.25. n = 90 obs. (66♀).

| **Response** | **Predictor** | **Link f.** | **Estimate** | **Std. Error** | **df** | **Crit. Value** | **P. Value** | **Std. Estimate** |
| --- | --- | --- | --- | --- | --- | --- | --- | --- |
| Estrus | Daily visits | Logit | **0.102** | 0.042 | 85 | 2.427 | 0.0152 | **0.446** |
| Estrus | Year 2023 | Logit | **4.441** | 0.913 | 85 | 4.863 | <0.001 | **0.733** |
| Estrus | Body mass | Logit | 0.101 | 0.054 | 85 | 1.868 | 0.0618 | 0.265 |
| Estrus | Daily visits: Year 2023 | Logit | **-0.124** | 0.052 | 85 | -2.377 | 0.0175 | **-0.457** |
| Body mass | Daily visits | Normal | **0.268** | 0.058 | 87 | 4.608 | <0.001 | **0.446** |
| Body mass | Year 2023 | Normal | 2.195 | 1.540 | 87 | 1.426 | 0.1575 | 0.138 |
